# Prior hormetic priming is costly under environmental mismatch

**DOI:** 10.1098/rsbl.2013.1010

**Published:** 2014-02

**Authors:** David Costantini, Pat Monaghan, Neil B. Metcalfe

**Affiliations:** 1 Institute for Biodiversity, Animal Health and Comparative Medicine, University of Glasgow, Glasgow G12 8QQ, UK; 2 Department of Biology, University of Antwerp, Wilrijk 2610, Belgium

**Keywords:** early life, environmental mismatch, heat stress, hormesis, survival

## Abstract

It is increasingly recognized that hormetic environmental priming of stress responses can improve resilience to later life stress exposure. However, such phenotypic adjustments may be costly, particularly if the subsequent environment does not match that to which the adjustment was made. Here, we show that hormetic priming to mild heat stress in early life increases survival only when heat stress is again experienced in adulthood; it reduces survival if the stressor is not encountered again. That such costs can occur explains both why the stress response system is not maintained in an upregulated state and why the hormetic adjustment of responses has evolved.

## 1. Introduction

A key issue in evolutionary ecology is whether phenotypic changes induced by early life experiences are, or are not, adaptive [1]. The environmental mismatch paradigm states that the extent to which a phenotypic adjustment is adaptive will depend on the degree to which early and adult environmental conditions are similar [1]. A change that is adaptive under certain developmental conditions may turn out to be maladaptive if the adult environment does not match that experienced in early life [1,2]. Hence, for early life phenotypic adjustments to be beneficial, the early environment needs to be predictive to some extent of the adult environment [1,2].

Exposure to stressful circumstances in early life can alter aspects of physiology; the long-term effects of such exposure are likely to depend both on the level of stressor experienced and on the adult environment. Exposure to severe stress in early life is generally detrimental to long-term health [2], but mild stress exposure can have a stimulatory and, possibly, beneficial effect through hormetic priming of stress response mechanisms, increasing the capacity to withstand exposure to a stressor later in life [3,4]. An important question then is why the need for such environmental priming has evolved, rather than having a system where the most effective responses can be expressed from the outset. The answer to this might lie with long-term costs associated with the stronger responses, costs which can be avoided if the adult environment is relatively stress free. Priming of stress responses in early life may therefore carry benefits for fitness providing the stressor is then encountered in the adult environment [5], but there might be a cost of phenotypic adjustments if there is no subsequent exposure to stress in adulthood. This may occur when the early life environment does not match the conditions experienced in adulthood. Although theoretical considerations support this view, we lack a clear experimental demonstration of the costs that early hormetic priming may carry for the individual under an environmental mismatch.

Using a small relatively short-lived songbird species (the zebra finch, *Taeniopygia guttata*), we tested the hypotheses that individuals exposed to mild heat stress earlier in life: (i) will live longer than individuals not pre-exposed to heat stress if heat stress is encountered in adulthood (reflecting the benefit of earlier priming of responses to heat stress) and (ii) will pay a penalty in terms of reduced survival if heat stress is not encountered again in adulthood (reflecting the costs of priming under environmental mismatch).

## 2. Material and methods

### (a) Experimental protocol

We used a factorial experimental design that allowed us to have four experimental groups, which varied with respect to early (control, mild stress) and adult (control, high stress) thermal experience in order to have a match or mismatch between the early and adult environment. Further details are given in the electronic supplementary material.

The thermal conditioning was started when birds were 42–45 days old. At this age, males and females can be distinguished by their plumage. Siblings were assigned randomly to two initial temperature treatment groups during the conditioning phase in early life: controls (C), kept throughout at the standard housing temperature and exposure to mild heat stress (S). Sexes were, however, not assigned randomly to the treatment groups, because we sought to keep a balanced sex ratio among groups. Temperatures were chosen according to [6]. Further details about the thermal conditioning are given in the electronic supplementary material.

In order to test how early exposure to stress affected survival, half of the birds of each early exposure treatment were allocated to each of two adult treatment groups at adulthood (177–180 days): control (C) or stress (S; 42°C—note that this is a more challenging thermal stress than in the early, priming, phase). Therefore, we had four experimental groups, which varied with respect to early and adult thermal experience: CC (12 males and 10 females), SC (12 males and 10 females), CS (12 males and nine females), SS (13 males and nine females). Birds were exposed to the short-term thermal adult treatment for 3 h every day for a total of 3 consecutive days, again being placed in the care units with ad libitum water but no food for the 3 h duration of each thermal treatment. At the end of the experiment, birds were maintained in their cages in the same conditions as before. The subsequent survival of the birds was then tracked for up to 3 years after the adult thermal challenge (the youngest and oldest birds still alive at the end of survival monitoring were 824 and 1099 days old, respectively). Over this period, no further manipulations were made to these birds. In captivity, zebra finch males typically live about 3 years and females about 2 years [7,8]. We therefore analysed survival over a timeframe relevant to evolutionary fitness in this species.

### (b) Statistical analyses

Cox mixed-effects models fitted by maximum likelihood in R (v. 3.0.1, R package ‘coxme’ mixed-effects Cox models) were used to test the effect of treatment on survival. We entered in the model two different factors for Treatment (early life Treatment and adult Treatment), plus Sex and the two- and three-way interactions. We included brood as a random factor to take into account any family effects, because there were siblings within our experiment. A censor variable was included to allow inclusion of adults still alive at the completion of the study. We used a backward elimination process to exclude independent variables with *p* < 0.05. Non-significant terms were sequentially removed from the models starting from the higher order interactions and the analyses were repeated until we obtained a model with only significant terms. The brood factor was always maintained in the models.

## 3. Results

**Figure 1.**
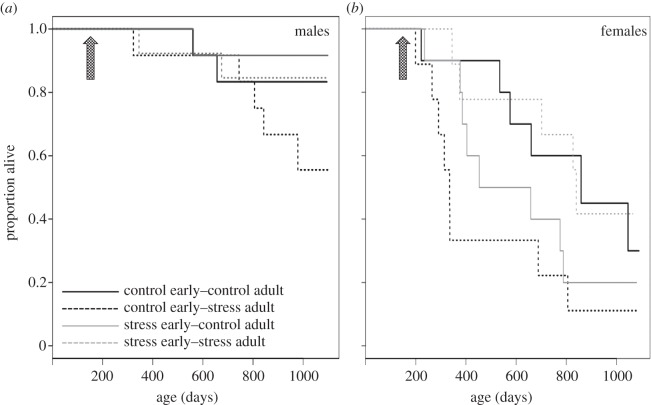
(*a*,*b*) Survival up to 3 years of age of control birds or those exposed to mild heat stress before sexual maturity in relation to their treatment in adulthood. The effects on survival of hormetic priming to mild heat stress in early life were contingent on whether heat stress was encountered again in adulthood. The timing of the adult temperature challenge is shown as an arrow.

The first deaths among females and males, respectively, were recorded 16 and 143 days after the end of the adult treatment. Both of these first birds to die were from the treatment group CS. Almost half of all the birds (45.9%) died over the first year following the adult treatment. The analysis of survival curves shows that the effects of hormetic priming to heat stress in early life were contingent on whether heat stress was or was not encountered again in adulthood (figure 1 and table 1). As predicted, survival was lower in those birds that (i) were controls in early life and were then exposed to heat stress in adulthood or (ii) experienced heat stress as young but not as adults. Overall, females survived less than males (table 1), but the response of males and females to the thermal treatment was generally similar across experimental groups (all two- and three-way interactions with sex, *p* ≥ 0.31; figure 1). The main difference between males and females in survival trends appears to occur for individuals that experienced mild heat stress in early life and were then controls in adulthood: while survival of females decreased with time, that of males did not do so (figure 1).

**Table 1.** Final outcome of a Cox mixed-effect model fitted by maximum likelihood showing the effects of early and late treatment on survival.

factor	*z*	*p*
early treatment	1.86	0.062
adult treatment	2.64	0.0083
sex	4.98	<0.001
early × adult	−2.37	0.018

## 4. Discussion

Our findings show that hormetic priming to heat stress would carry benefits for survival only if there is some degree of matching between the earlier and subsequent environments. When encountering relatively warm environments in adulthood, those birds that experienced heat stress earlier in life survived better than those that had not had the early life exposure, and their survival was similar to those birds not encountering heat stress at all. This demonstrates that early life stress exposure can be beneficial when there is environmental matching. However, when no heat stress was encountered in adulthood, survival was poorer in birds that experienced heat stress in early life than in those that did not do so; this demonstrates a cost of priming in the absence of a challenge in adulthood.

The cause of death was not the exposure to the heat stress itself. Zebra finches are tropical birds, and the temperature experienced was not outside their range of thermal tolerance [6]. However, it seems that the phenotypic adjustments to physiology as a consequence of the early life experience of heat stress do carry costs.

The reason for our focus on ambient temperature is not only the ease of implementing heat stress protocols, but also because high temperatures activate evolutionarily highly conserved stress response pathways [4,9]. Previous reports showed that manipulation of heat stress may have beneficial effects on longevity, but a recent meta-analysis on life-extension promoted by thermal hormesis suggests that any beneficial effect is dependent on the precise conditions of the initial priming: the likelihood of lifespan extension was greater if the initial priming was less severe, with shorter and less frequent heat shocks and longer recovery periods [10]. None of these earlier studies, however, considered the existence of a cost of hormetic priming as we have done in this experiment.

The results of our study also highlight the importance of elucidating the mechanisms that may explain the outcomes of early life priming. We do not know the actual cause of death of the birds in this study, but it was not due to injury or infection. We have previously proposed a role for oxidative stress in this context [11]: we found that mild heat stress experienced early in life primes zebra finches to better withstand oxidative stress induced by exposure to high ambient temperature in adulthood. By contrast, birds that had no previous experience of heat stress showed a significant increase in plasma oxidative damage when exposed to heat in adulthood and also exhibited a bigger decrease in red blood cell thiol antioxidants than the mild conditioning group [11]. Importantly, birds that experienced mild heat stress in early life and were then not exposed again to heat stress showed a decrease in thiols. This is relevant because a disruption of homoeostatic mechanisms through overoxidation of thiols makes the cells less capable of efficiently controlling the activity of free radicals [12,13]. Hence, a cost of hormetic priming may come through a long-term impairment in the regulation of mechanisms that control oxidative balance. It might be that the resources required to maintain a molecular ‘memory’ needed to withstand stressful episodes become a significant cost when such events do not occur, if these resources are taken away from other organism functions that may impact on survival. The birds that we used in our study were fully grown at the time that the priming occurred, but still immature. To what extent the results we observed are dependent upon the priming having taking place relatively early in life is unclear, though a number of studies suggest that early life is a particularly sensitive stage [3,4]. It is possible that the results could have been more marked if the birds had been even younger when the priming took place, but also possible that priming in adulthood could have a similar effect.

In conclusion, our study shows for the first time that environmentally primed stress resistance in early life may carry survival costs if the early environment is not predictive of the stress load of the adult environment. This may explain both why the stress response system is not maintained in an upregulated state and why the hormetic response has evolved. Future challenges include determining the mechanisms that underlie these survival costs under environmental mismatch.

## Data accessibility

Raw data can be found in the electronic supplementary material.

## References

[1] Monaghan P 2008 Early growth conditions, phenotypic development and environmental change. Phil. Trans. R. Soc. B 363, 1635–1645 (doi:10.1098/rstb.2007.0011) 18048301 10.1098/rstb.2007.0011 PMC2606729

[2] Harris A Seckl J 2011 Glucocorticoids, prenatal stress and the programming of disease. Horm. Behav. 59, 279–289 (doi:10.1016/j.yhbeh.2010.06.007) 20591431 10.1016/j.yhbeh.2010.06.007

[3] Costantini D Metcalfe NB Monaghan P 2010 Ecological processes in a hormetic framework. Ecol. Lett. 13, 1435–1447 (doi:10.1111/j.1461-0248.2010.01531.x) 20849442 10.1111/j.1461-0248.2010.01531.x

[4] Mattson MP Calabrese EJ 2010 Hormesis: a revolution in biology, toxicology and medicine. New York, NY: Springer

[5] Sheriff MJ Love OP 2013 Determining the adaptive potential of maternal stress. Ecol. Lett. 16, 271–280 (doi:10.1111/ele.12042) 23205937 10.1111/ele.12042

[6] Calder WA 1964 Gaseous metabolism and water relations of the zebra finch, *Taeniopygia guttata*. Physiol. Zool. 37, 400–413

[7] Burley N 1985 Leg-band colour and mortality patterns of captive breeding populations of zebra finches. Auk 102, 647–651

[8] Nager RG Law G 2010 *The zebra finch* In The UFAW handbook on the care and management of laboratory and other research animals (eds Hubrecht R Kirkwood J), 8th edn., pp. 674–685 Oxford, UK: Wiley-Blackwell

[9] Rattan SIS 2008 Hormesis in aging. Ageing Res. Rev. 7, 63–78 (doi:10.1016/j.arr.2007.03.002) 17964227 10.1016/j.arr.2007.03.002

[10] Lagisz M Hector KL Nakagawa S 2013 Life extension after heat shock exposure: assessing meta-analytic evidence for hormesis. Ageing Res. Rev. 12, 653–660 (doi:10.1016/j.arr.2013.03.005) 23570942 10.1016/j.arr.2013.03.005

[11] Costantini D Monaghan P Metcalfe N 2012 Early life experience primes resistance to oxidative stress. J. Exp. Biol. 215, 2820–2826 (doi:10.1242/jeb.072231) 22837454 10.1242/jeb.072231

[12] Jones DP 2006 Redefining oxidative stress. Antiox. Redox Signal. 8, 1865–1879 (doi:10.1089/ars.2006.8.1865) 10.1089/ars.2006.8.1865 16987039

[13] Sohal RS Orr WC 2012 The redox stress hypothesis of aging. Free Rad. Biol. Medic. 52, 539–555 (doi:10.1016/j.freeradbiomed.2011.10.445) 10.1016/j.freeradbiomed.2011.10.445 PMC3267846 22080087

